# Determinants of health-related quality of life in elderly in Tehran, Iran

**DOI:** 10.1186/1471-2458-8-323

**Published:** 2008-09-22

**Authors:** Maryam Tajvar, Mohammad Arab, Ali Montazeri

**Affiliations:** 1 London School of Hygiene and Tropical Medicine, London, UK; 2 Department of Health Management and Economics, Tehran University of Medical Sciences, Tehran, Iran; 3 Iranian Institute for Health Sciences Research, Tehran, Iran

## Abstract

**Background:**

As Iran started to experience population ageing, it is important to consider and address the elderly people's needs and concerns, which might have direct impacts on their well-being and quality of life. There have been only a few researches into different aspects of life of the elderly population in Iran including their health-related quality of life. The purpose of this study was to measure health-related quality of life (HRQoL) of elderly Iranians and to identify its some determinant factors.

**Methods:**

This was a cross-sectional survey of a random sample of community residents of Tehran aged 65 years old and over. HRQoL was measured using the Short From Health Survey (SF-36). The study participants were interviewed at their homes. Uni-variate analysis was performed for group comparison and logistic regression analysis conducted to predict quality of life determinants.

**Results:**

In all, 400 elderly Iranian were interviewed. The majority of the participants were men (56.5%) and almost half of the participants were illiterate (n = 199, 49.8%). Eighty-five percent of the elderly were living with their family or relatives and about 70% were married. Only 12% of participants evaluated their economic status as being good and most of people had moderate or poor economic status. The mean scores for the SF-36 subscales ranged from 70.0 (SD = 25.9) for physical functioning to 53.5 (SD = 29.1) for bodily pain and in general, the respondents significantly showed better condition on mental component of the SF-36 than its physical component (mean scores 63.8 versus 55.0). Performing uni-variate analysis we found that women reported significantly poorer HRQoL. Multiple logistic regression analysis showed that for the physical component summary score of the SF-36, age, gender, education and economic status were significant determinants of poorer physical health-related quality of life; while for the mental component summary score only gender and economic status were significant determinants of poorer mental health-related quality of life. The analysis suggested that the elderly people's economic status was the most significant predictor of their HRQoL.

**Conclusion:**

The study findings, although with a small number of participants, indicate that elderly people living in Tehran, Iran suffer from relatively poor HRQoL; particularly elderly women and those with lower education. Indeed to improve quality of life among elderly Iranians much more attention should be paid to all aspects of their life including their health, and economic status.

## Background

We live in a 'population ageing' era. Population ageing has progressed furthest in developed countries but developing countries have also begun to experience considerable increases in their proportion of elderly people [1,2]. Iran has started to come across with the population ageing phenomenon too. Although, Iran still has a relatively young population, the proportion of elderly is projected to double in less than 20 years [3]. The United Nations statistical projections demonstrate rapid growth of elderly population in Iran. While the proportion of people with 60 years old age and above in Iran was 5.4 percent in 1975 it will increase to 10.5 percent in 2025 and 21.7 percent in 2050 [4]. In fact the total size of population of Iran will fail to double in the next fifty years, but the number of elderly aged 65 years and over will experience about six-fold increase [5]. Thus, it is no longer possible to ignore the commencing ageing phenomenon in Iran and therefore, it is vital to anticipate requirements of this age group in Iran to plan appropriate policies to address their growing needs and to support their quality of life.

In Iran the elderly are treated very respectfully and they are privileged by a high position among the family members and are supported by their family for all their needs. The Islam also supported this belief where 95% of the general populations and 99.2% of the elderly are Muslim [6,7]. There are several verses in the Quran stating that Muslims should appreciate and regard the elderly as valuable and precious members of community (e.g. Verse 23 of Asra Surah, Quran). There are also many poems and expression in Persian literature regarding the respected position of elderly in families and in the community as the builders of our past and the repository of life experiences. In Iran, while considerable decline appears in the health of the elderly through getting older, their ability to obtain their health needs seems even getting worse than before. It happens because most of people loose their income source in ageing period and become economically dependent to others. On the other hand, medical expenses and prices increase every year in Iran and this issue deteriorates the ability of elderly to pay for their medical needs particularly for those lacking any medical insurances [8]. According to a survey, 25 to 30% of the elderly in Iran did not benefit from any medical insurance services [7]. Therefore, thousands of old men and women are likely to face further hazards for their health in Iran. However, only a few studies have been conducted in Iran on different aspects of life of the elderly including their health-related quality of life (HRQoL).

HRQoL is a subdivision of QoL, and most commonly refers to people's experience of their global health. It may also refer to health-related subjective well-being, functional status, or self-perceived health [9,10]. A representative definition of HRQoL is "a multi-dimensional concept that encompasses the physical, emotional, and social components associated with an illness or treatment" [11]. However, health related quality of life for the elderly people might be described in terms of functional status, independence and ability to engage in life activities [12]. An important aim of research into HRQoL in this age group is to enable older people to maintain their mobility, independence, their active contribution to society, and to respond effectively to the challenges of older age and in general bring an active aging for them [13]. It is argued that the increasing international interest in research into HRQoL is partly due to global population ageing, as a longer life is often associated with a higher proportion of chronic diseases and functional impairments [7]. The elderly with chronic disorders often experience a burden of diseases that adversely influence their HRQoL [5]. Therefore, investigating HRQoL of the elderly is especially important because health issues limit their independence and ability to engage in life activities.

Health-related quality of life and its determinants in older people is well documented in developed world. For instance, a study by Gallicchio et al. [14] showed that poor social networks are associated with worse physical health and mental well-being. Other factors such as living in poor housing, inadequate finances and inadequate social relationships were also important factors leading to deterioration in QoL [13]. Farquhar [15] reported that older people identified family relationship, health, standard of living, activities and other social contacts important to bring quality to their life. Bowling [13] also revealed that good health, good social relationship, having social activities, good financial circumstances and being independent significantly would increase QoL in elderly populations. However, health-related quality of life and its determinants in elderly are not researched adequately in Iran. Thus, this study sought to assess HRQoL in a sample of elderly of Tehran in order to identify some of its contributing factors. Understanding the factors contributing to HRQoL is critical for developing the most appropriate interventions for improving or preserving QoL. In this study we examined the association between several important characteristics of elderly people in Tehran including sex, age, education, living status, marital status and economic status with HRQoL. We were interested to understand whether these variables are the significant predictors for HRQoL in the elderly people or not. We hoped that the result of this research could effectively contribute to the challenges of people with older age in Iran.

## Methods

### Study design and data collection

This was a cross-sectional survey of a random sample of elderly Iranians selected from the general population in Tehran. Tehran has more than 7 million inhabitants and 22 districts and it is most densely populated region in Iran [16]. The sampling method was based on a multi-stage stratified sampling approach. Information on the total number of households and their addresses were available for all districts (provided by the municipality of Tehran). Proportionate allocation sampling was used to identify a sampling fraction for each of the districts. Then, random sampling was applied within each stratum to select the required households in the districts to ensure that every household within the districts has the same probability of being sampled. All participants were interviewed at their home. Those who were not available for interview at given time were asked for another appointment. Twelve interviewers were trained to collect the data.

### Measure of health-related quality of life

We used the Iranian version of Short Form Health Survey (SF-36) questionnaire to collect data on HRQoL. The SF-36 is a well-known generic HRQoL instrument that was developed initially in the United State of America. Its reliability and validity has been approved not only in multiple populations in several studies, but also for elderly people in some surveys [17]. The psychometric properties of the Iranian version of the SF-36 (interview administered) are well documented [18].

The SF-36 includes 8 subscales namely: Physical functioning (PF), Role physical (RP), Bodily pain (BP), General health (GH), Vitality (VT), Social functioning (SF), Role emotional (RE), and Mental health (MH). It also provides two summary scales, Physical Component Summary (PCS) and Mental Component Summary (MCS). Scores range from 0 to 100 for each subscale with higher scores indicating a better condition.

### Independent variables

The other data collected were included sex, age, education, living status, marital and economic status. The *living status *of the elderly was represented by two main categories living alone or with others. *Marital status *was categorized into two main subgroups; married and non-married people. *Education *was categorized into three groups: illiterate, middle literacy, and university education. This was done due to the fact that illiteracy and university education have significant impact on the health status of elderly people in Iran, while people with an education level between the two mentioned levels are usually the same (although too broad). Thus, all people other than the two indicated groups were catagroized as "middle literacy" group as we believed that they may not have significant socioeconomic differences considering Iran's current condition. Finally, the *economic status *of people was indicated by asking each individual to respond to the question "In general, how would you describe your economic status at present"? There were three response categories: poor, intermediate, and good. It has been shown that this is generally a reliable method to collect such information as people are able to accurately rate their economic status with respect to the community condition and compare themselves with others [19].

### Data analysis

The data were analyzed using both descriptive and analytical approaches. The normality of the data (the SF-36 scores) was examined. Although score distributions slightly were negatively skewed, all were found to be satisfactory (all skewness values less than one). To examine the association between the participants' characteristics and their HRQoL, uni-variate statistical tests including T-Tests and One-way analysis of variance (ANOVA) were performed. To indicate determinant factors of HRQoL multiple logistic regression analysis was applied. For the purpose of the logistic regression analysis Physical Component Summary (PCS) and Mental Component Summary (MCS) were used and relative to the mean scores the study sample were divided into two groups; those who scored equal or greater than mean (PCS: n = 206; MCS: n = 227) and those who scored below mean (PCS: n = 194; MCS: n = 173).

### Ethical considerations

This study received approval from the ethics committee of Tehran University of Medical Sciences (TUMS). All participants gave their oral consents for interview. We kept the information of the participants confidential.

## Results

In all, 400 elderly Iranians aged 65 years and over were interviewed. As shown in Table 1, 56.5% of the participants were men. The mean age of participants was 72 years (SD = 6.3). Only a few people, 4.5%, had university education and approximately half were illiterate. Most were living with family or relatives (85%) at the time of interview. 62.7% were married. Of total participants, 35.8% described their own economic status poor, 52.2% moderate and the remaining 12% good.

**Table 1:** Frequency distribution of the participant's demographical characteristics (n = 400)

		Number	Percent (%)
Sex			
	Female	174	43.5
	Male	226	56.5
Age			
	65–69	153	38.3
	70–74	126	31.5
	75–79	61	15.3
	80+	60	15.0
Living status			
	Alone	60	15.0
	With others	340	85.0
Education			
	Illiterate	199	49.8
	Middle literacy	183	45.8
	University education	18	4.5
Marital Status			
	Married	279	69.8
	Non-married	121	30.2
Economic Status			
	Poor	143	35.8
	Intermediate	209	52.2
	Good	48	12

Table 2 presents HRQoL scores as measured by the SF-36. The mean (SD) of physical and mental summary scores were 55.01 (25.66) and 63.86 (23.86) respectively; indicating that the mental status of the participants was significantly better than their physical condition (P < 0.0001).

**Table 2:** Mean scores of HRQoL of elderly in Tehran as measured by the SF-36

Scales	Means	SD
Physical functioning (PF)	54.96	30.65
Role physical (RP)	56.37	48.18
Bodily pain (BP)	53.59	29.10
General health (GH)	55.11	21.06
Vitality (VT)	55.87	24.08
Social functioning (SF)	70.93	25.93
Role emotional (RE)	65.50	45.68
Mental health (MH)	63.14	22.33
Physical Component Summary (PCS)	55.01	25.66
Mental Component Summary (MCS)	63.86	23.86

The association between the participant's socio-demographic characteristics and their HRQoL was also examined. Table 3 summarizes the results.

**Table 3:** Association between the SF-36 scores and socio-demographical characteristics of the study sample

Scales	Sex		P value	Age Groups				P-value	Marital Status		P-value
				
	Male mean (SD)	Female mean (SD)		65–69 mean (SD)	70–74 mean (SD)	75–79 mean (SD)	80+ mean (SD)		Married mean (SD)	Unmarried mean (SD)	
PF	62(29)	45.7(30.2)	0.000	62.6(30.2)	55.4(29.5)	48.5(29.6)	40.9(29.3)	0.001	59.1(29.5)	45.2(31.1)	0.000
RP	62.3(47)	48.5(48.5)	0.004	69.2(44.8)	54.9(48.9)	38.9(47.5)	44.1(47.2)	0.000	61.4(46.9)	44.6(49)	0.001
BP	62.3(27.7)	42.1(26.7)	0.000	57(28.9)	52.3(27.5)	51.5(32.3)	49.5(28.9)	0.105	58.1(28.4)	43(27.8)	0.000
GH	59.9(19.4)	48.7(21.4)	0.000	57.1(19.9)	53.8(21.8)	53.7(22)	54(21.1)	0.409	57.2(20.5)	50.1(21.3)	0.002
VT	62.2(21.4)	47.5(24.8)	0.000	58.8(24.1)	55.1(23.5)	52.4(22.6)	53.2(26)	0.394	58.7(22.8)	49.7(25.7)	0.000
SF	74.8 (23.9)	65.8(27.5)	0.001	72.4(25)	71.9(25.5)	67.6(27.6)	68.3(27.3)	0.264	73.3(24.6)	65.2(28)	0.004
RE	72.2(42.8)	56.7(47.8)	0.001	68.8(45.3)	63.4(46.2)	61.7(46.6)	65(44.8)	0.154	76(45)	61.9(47.2)	0.311
MH	67(20.7)	58(23.3)	0.000	64.8(21.8)	62.7(22.9)	60.6(23.2)	62.3(21.5)	0.475	65.5(21.4)	57.6(23.3)	0.001
PCS	61(24.1)	46.3(25)	0.000	61.5(24.7)	54.1(25.1)	48.1(26.6)	47.1(24.4)	0.058	59(24.8)	45.7(25.2)	0.000
MCS	69.1(22)	57(24.4)	0.000	66.2(23.2)	63.3(24.6)	60.6(24.7)	62.2(23)	0.655	66.1(23)	58.5(25)	0.003

Scales	Living status		P value	Education			P-value	Economic Status			P-value
	Alone mean (SD)	With others mean (SD)		Illiterate mean (SD)	Middle literacy mean (SD)	University education mean(SD)		Poor mean (SD)	Intermediate mean (SD)	Good mean (SD)	

PF	43.4(33)	57(29.8)	0.001	46.5(29.3)	63.1(29.6)	64.4(30.6)	0.000	45.1(31.7)	58(27.9)	72(28.8)	0.000
RP	42(49.4)	58.8(47.5)	0.013	47.1(48.7)	65.9(46)	61.1(46.3)	0.001	39(47.2)	63.1(46.7)	79.1(41)	0.000
BP	43.6(28.5)	55.3(28.8)	0.004	45.7(27.4)	60.6(28.8)	68.3(26.2)	0.000	46.7(28.1)	56.4(28.8)	62(29.9)	0.000
GH	48.4(20.8)	56.3(20.9)	0.007	50.1(21.1)	60.1(19.7)	59.7(21.1)	0.000	47.5(19.8)	58.5(20.7)	63.4(19.2)	0.000
VT	50.8(24.9)	56.7(23.8)	0.079	49.3(22.1)	62(24.3)	65.2(23.4)	0.000	47.8(23)	58.5(23.2)	69.2(22.2)	0.000
SF	61.6(28.6)	72.5(25.1)	0.003	66.7(25.1)	75(26.2)	75.6(25.1)	0.005	62.1(26.9)	74.9(24)	79.9(23.5)	0.000
RE	61.1(47.9)	66.2(45.2)	0.420	56.9(47.5)	73.2(42.71)	81.4(36.55)	0.001	50.9(48.4)	72.1(42.7)	80.5(39.4)	0.000
MH	58.9(20.9)	63.8(22.5)	0.114	57.4(22)	68.9(20.9)	66.8(24)	0.000	55.6(20.8)	66(28.8)	74(21.5)	0.000
PCS	44.4(26.9)	56.8(25)	0.000	47.3(24.9)	62.4(24.5)	63.4(19.6)	0.000	44.6(25.5)	59(23.6)	69.2(23)	0.000
MCS	58.1(24.9)	64.8(23.5)	0.044	57.6(22.8)	69.8(23.5)	72.3(20)	0.000	54.1(23.5)	67.9(22.2)	75.9(21.4)	0.000

There were significant differences between men and women on all the SF-36 and the PCS and the MCS scores indicating that older women reported significantly poorer HRQoL compared with men (P < 0.0001). Scores were not significantly different among age groups but for the physical functioning and the role physical (P < 0.0001). The results also demonstrated that there were statistically significant differences in HRQoL of the respondents in terms of their educational level (P < 0.0001). Higher education was associated with better HRQoL in all aspects of the SF-36. The elderly living with others had a higher average in all HRQoL scales compared to people living alone (P < 0.0001). However, this difference was not statistically significant for vitality, role emotional and mental health subscales. The married elderly living with their spouse had higher HRQoL scores compared with those who were not married (P < 0.000). Furthermore, the elderly enjoying by a high economic status in the community had higher HRQoL scores (P < 0.0001).

To indicate determinant factors of HRQoL, multiple logistic regression analysis was performed. As indicated in Table 4, the results showed that for the PCS age, sex, education and economic status were significant determinants. The results showed no significant results for living condition and marital status either. However, for the MCS the results showed a relatively different perspective indicating that only sex and economic status were significant determinants. Age, marital status, education and living condition did not show significant results although the findings were in the expected direction.

**Table 4:** Determinants of poor physical and mental health-related quality of life in elderly participants

	OR (95% CI)	P
Physical Component Summary (PCS)		
Age		
65–69	1.0 (ref.)	
70–74	1.48 (0.88–2.48)	0.13
75–79	2.36 (1.21–4.59)	0.01
80 ≥	2.62 (1.33–5.14)	0.005
Sex		
Male	1.0 (ref.)	
Female	2.42 (1.44–4.04)	0.001
Marital status		
Married	1.0 (ref.)	
Never married/widowed/divorced	1.31 (0.70–2.43)	0.39
Education		
Literate	1.0 (ref.)	
Illiterate	1.52 (0.96–2.41)	0.07
Economic status		
Good	1.0 (ref.)	
Intermediate	1.76 (0.85–3.64)	0.12
Poor	4.0 (1.83–8.70)	< 0.0001
Living condition		
With family/relatives	1.0 (ref.)	
Alone	1.05 (0.49–2.24)	0.89
Mental Component Summary (MCS)		
Age		
65–69	1.0 (ref.)	
70–74	1.25 (0.75–2.10)	0.38
75–79	1.29 (0.68–2.48)	0.42
80 ≥	1.47 (0.76–2.83)	0.24
Sex		
Male	1.0 (ref.)	
Female	2.48 (1.50–4.12)	< 0.0001
Marital status		
Married	1.0 (ref.)	
Never married/widowed/divorced	0.99 (0.54–1.83)	0.99
Education		
Literate	1.0 (ref.)	
Illiterate	1.20 (0.75–1.89)	0.43
Economic status		
Good	1.0 (ref.)	
Intermediate	2.26 (1.06–4.81)	0.03
Poor	4.85 (2.18–10.8)	< 0.0001
Living condition		
With family/relatives	1.0 (ref.)	
Alone	1.07 (0.51–2.21)	0.85

The analysis suggested that the elderly people's economic status was the most significant predictor of a better or a poorer physical and mental health-related quality of life scores.

## Discussion

In general, based on the findings of the present study we might conclude that HRQoL in participants, particularly physical health, was rather poor; although the study sample was small and the results could not be generalized to entire elderly population in Iran. To explain such findings one might argue that most elderly Iranian are poor and often their income does not adequately cover their living expenses and thus the elderly, particularly women, face multiple problems that influence the quality of their life [20,21]. The results of the first national survey in 1998 on health and ageing in Iran showed that the employment rate was 42–64% among elderly males while just 2.7–9.3% of females were paid employees. Urban seniors paid more visits to physicians compared with rural seniors; 20–25% experienced a trauma in a year that could lead to special therapeutic and medical measures, 25% of urban and 35% of rural elderly needed eyeglasses or lenses, and more than 50% of elderly people had a disability on moving and transfer, such as arthritis or osteoporosis [7]. In addition, one might attribute the findings to poor health care services for elderly people compared to the general population due to several factors including economic barriers [22].

We found that elderly people in this study had a better mental health condition compared to their own physical health. This might reflect the socio-cultural position that elderly people poses in Iran. The dominant Iranian culture places a high position for old people among the family members and relatives and embeds a good social relationship between young and old members. The high score for social functioning subscale may also support this explanation.

Examination of the associations between HRQoL subscales and socio-demographical factors demonstrated clear patterns. We observed that women had significantly poorer HRQoL in all scales compared with men. All available studies on HRQoL in Iran without exception obtained the same results that can be interpreted as significant gender inequalities in health in Iran [18,22,23]. In general Iranian women (particularly old women) have less access to information, education, and employment and in overall disadvantaged economic status and social position compared with men. This cause a weaker access to resources and decision-making positions which constrain women's ability to influence resource allocation, investment and expenditure decisions [24]. All the above matters definitely generate a worse health status and HRQoL for women. Similarly studies of HRQoL worldwide including Finland, Taiwan, Poland and Croatia, Japan and Korea have found that women were less advantaged group compared to male group [25-29]. Although women on average live longer than men, they report more illness than men [30]. Estimates of healthy life expectancy from 2002 showed that in almost all countries women have fewer healthy years of life than men [31]. The condition for Iranian women is in average even worse than condition for women in rest of the world. The lower proportion of females than elderly males in Iran in comparison with other countries also reflects the disadvantaged life conditions for females. Improving women's health and their HRQoL demands a multi-sectional, multi-disciplinary, and culturally relevant approach to create a suitable environment for providing better living conditions for women.

We observed that age, not surprisingly, negatively affects the HRQoL mostly on physical health than mental health. It, however, have been discussed that if elderly have satisfactory living conditions, increasing age may not result in deterioration of their mental health [32,33]. Thus, the attempts should be made to delay or limit the impact of age on the body in order to give higher quality to the life of the aging population.

A strong association was observed between education and HRQoL. According to Lasheras [34] lower educational level is associated with unhappiness, poor social relationships, poor self-assessed health, and sensory problems among the elderly. Education is an important indicator that may directly or indirectly influence HRQoL through its association with higher social class and economic status [35].

The present study also showed a better HRQoL for the elderly living with others compared to those living alone. Likewise, married people enjoyed by a higher HRQoL than widowed, single and divorced elderly. The study by Vahdainia et al. [23] showed that elderly living with their spouse in Tehran had better HRQoL in all 8 subscales of the Sf-36 compared with those who were widowed or divorced. Victor et al. [36], and Walker [37] argued that low amount of social participation and being alone is often associated with poor HRQoL in old ages. According to the study by Bowling et al. [38] poor psycho-social health and feelings of loneliness has been seen among those living alone due to lack of emotional support within the household, and an absence of practical support. However, others presented different perspective and argued that living alone is not necessarily the same as feeling loneliness and experience a poor QoL [36,39]. Some researchers believe that poor health is often a reason for moving to live with a relative. Therefore, the elderly who live with relatives may have poorer health than those living alone [13]. However, differences in the above perspectives reflect diversity in cultural and social conditions of communities.

In Iran, the cultural and religious background is not in favor of leaving elderly people alone and encourages younger people to take care of their elderly parents; while in developed societies often older people value their independence and may prefer to live alone [38]. In Iran the care of elderly people in nursing homes or institutions are largely deemed unacceptable by the general public. However, due to recent changes of family size, migration and accommodation problems, there is a trend to transfer elders to nursing homes for better care [7].

Last but not least, economic status recognized as the most important predictor of HRQoL of elderly among other factors examined. Having enough money is important to QoL, not only to cover and meet the basic needs of life, but it is a very influential factor to participate in society, to enjoy people themselves of hobbies, holidays and luxuries and to make elderly free of worry about emergencies in life and unexpected expenses in future [13]. This is one of the reasons that elderly Iranians have considerably lower HRQoL scores compared with the scores of elderly in developed world. Omnibus survey in the UK demonstrated considerably better scores both physically and mentally for British elderly [40].

Other determinants of QoL in older people, were identified as social networks, standards of living, activity, spirituality, material resources and physical and social environmental factors [9]. In addition, people's expectation of life, optimism or pessimism, having good health and physical functioning, participating in social activities and having social and family supports, good community services such as transport, safety and having control on their own life were found to be important elements of health-related quality of life in elderly people [41].

This study, however, has several limitations. The study design was cross-sectional and it is hence difficult to establish cause-effect relationships between QoL and socio-demographic characteristics. A longitudinal study is needed to investigate the relationships in the future. Our sampling took into account only non-institutionalized individuals and excluded those living in nursing homes, hospitals for the chronic diseases. As such the design might have biased our results in a way that we recruited a sample of better off elderly people. Another limitation of this study is that we collected the data via face-to face interviews by 12 interviewers rather than self-reporting method. Thus, although interviewers were trained, there are possibilities that they might be collected the data differently. In addition, some elderly might be either 'under-reported' or 'over-reported' their QoL depending on the time and place of interviews. Social desirability bias, for instance, may cause some to over-report their QoL. This bias is likely to be stronger in a face-to-face interview compared to a self-report questionnaire [42]. Mood states of people at the time of answering QoL questions also can affect responses [37]. However such limitations should be minimized in the future studies. Further studies could also be completed to identify the likely causes of inequalities in health in terms of gender, living status and socio-economic position. Additionally, qualitative studies such as in-depth interviews with elderly can be used to have a better understanding on the topic.

## Conclusion

The study findings indicate that elderly people living in Tehran, Iran suffer from relatively poor HRQoL; particularly elderly women and those with lower education. Indeed to improve quality of life among elderly Iranians much more attention should be paid to all aspects of their life including their health, and economic status. It is hoped that this survey could add to the existing literature on HRQoL of old people in Iran and enable informed decisions to be made by policy makers.

## Abbreviations

QoL: Quality of life; HRQoL: Health-related quality of life; SF-36: Short-Form 36 Health Survey; PCS: Physical Component Summary; MCS: Mental Component Summary; GH: General Health; MH: Mental Health; PF: Physical Functioning; BP: Bodily Pain; RE: Role Emotional; RP: Role Physical; SF: Social Functioning; VT: Vitality.

## Competing interests

The authors declare that they have no competing interests.

## Authors' contributions

MT was the principal investigator and wrote the paper. MA was also the principal author of this paper. He managed the study throughout the work. He contributed equally to this work with MT. AM has made substantial contributions to the analysis and interpretation of the data. He also reviewed the first draft and wrote the final version of the manuscript. All authors read and approved the final manuscript.

## Pre-publication history

The pre-publication history for this paper can be accessed here:


